# Tumor protein 53 mutations are enriched in diffuse large B-cell lymphoma with irregular CD19 marker expression

**DOI:** 10.1038/s41598-017-01800-6

**Published:** 2017-05-08

**Authors:** Marina Kazantseva, Noelyn A. Hung, Sunali Mehta, Imogen Roth, Ramona Eiholzer, Alison M. Rich, Benedict Seo, Margaret A. Baird, Antony W. Braithwaite, Tania L. Slatter

**Affiliations:** 1 Department of Pathology, Dunedin School of Medicine, University of Otago, Dunedin, New Zealand; 2 Sir John Walsh Research Institute, Faculty of Dentistry, University of Otago, Dunedin, New Zealand

## Abstract

Accumulating evidence suggests tumor protein 53 (p53) promotes correct cellular differentiation. Thus, mutant *TP53* may be more frequent in tumors with irregular differentiation. This study investigated whether *TP53* mutations were more frequent in diffuse large B cell lymphoma (DLBCL) that lacked the B cell lineage marker CD19. Sixteen CD19 negative and 78 CD19 positive DLBCL were sequenced for *TP53* mutations. Twenty nine tumors had *TP53* mutations and were associated with poorer survival. Mutant *TP53* was more frequent in CD19 negative lymphomas (81% versus 21%, p < 0.0001). Analysis of other B cell markers revealed a lack of paired box 5 (PAX5) in CD19 positive lymphomas with mutant *TP53* (50%), which was more frequent compared to tumors with wild-type *TP53* (15%, p = 0.002). In summary, DLBCL lacking CD19 or PAX5 expression were more likely to have mutant *TP53*, suggesting irregular B cell marker phenotypes are associated with *TP53* mutation.

## Introduction

Normal functioning of p53 is critical for preventing cancers. We know this because mice with defects in *Trp53* are highly tumor prone; in humans inherited *TP53* mutations, Li-Fraumeni Syndrome, is characterized by multiple tumor types, and about half of all human cancers contain *TP53* mutations (www.iarc.p53.fr) [reviewed in ref. 1]. The nature of *TP53* mutations may provide clues as to the molecular events associated with the development of particular types of cancer^2^.

p53 is a transcription factor that prevents cancers forming by responding to DNA damage and initiating signaling pathways that repair DNA, permanently arrest cell division, or those that induce apoptosis to permanently remove damaged cells [reviewed in ref. 1]. As well as preventing cancer, normal functioning of p53 is required for many biological processes including cell differentiation. In hematopoietic cells, the introduction of wild-type p53 into transformed mouse pre-B cells induced B cell maturation^3^ and mice deleted for the proline-rich domain (PRD) of p53 develop B cell lymphomas comprised of incorrectly differentiated (CD19 negative) B cells^4^.

Diffuse large B cell lymphoma (DLBCL) is the most common and aggressive type of non-Hodgkin lymphoma, with *TP53* mutations present in approximately 20% of tumors^5, 6^. A poorer prognosis is associated with DLBCL with mutant p53^5–10^. Of interest, 10–12% of DLBCL have lost the B cell lineage marker CD19^11, 12^ and are also associated with a poor prognosis^5, 6, 12^. Given this, and the data from the PRD mutant mouse, we were interested to determine if mutant p53 might be driving abnormal B cell differentiation that pre-disposes to malignancy. This study investigated the prevalence of *TP53* mutations in DLBCL with irregular expression of B cell markers including those that were CD19 negative.

## Results

### *TP53* mutations are enriched in CD19 negative DLBCL

**Figure 1:**
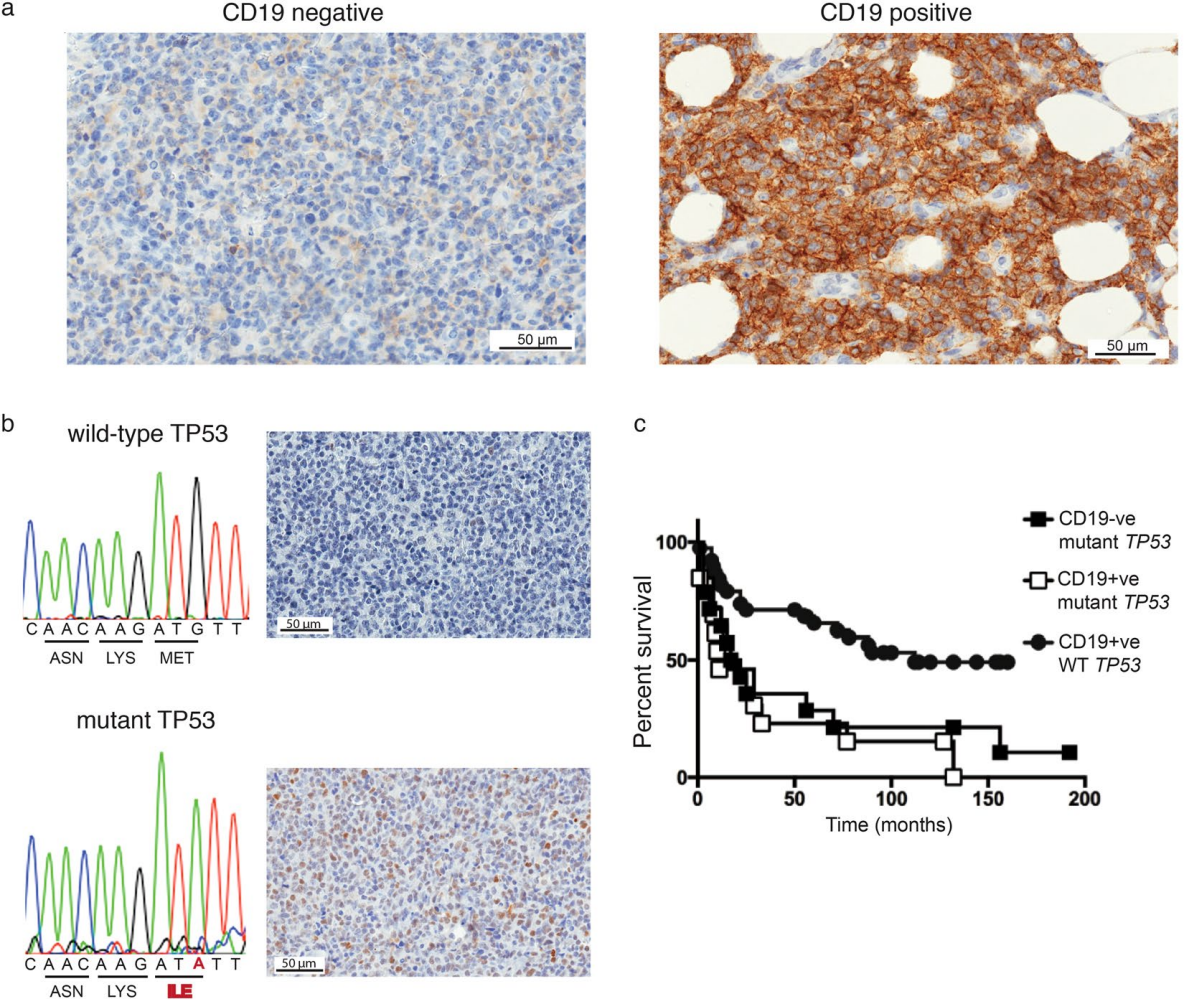
Tumor protein 53 mutations in CD19 negative DLBCL. (**a**) Left panel, CD19 negative lymphomas lacked CD19 staining using immunohistochemistry. Right panel, CD19 positive lymphomas showed strong CD19 staining. (**b**) Lymphomas were sequenced to identify *TP53* mutations. Sequence analysis of a CD19 negative lymphoma with a M133I mutation is given in comparison to the wild-type *TP53* sequence. Increased p53 by immunohistochemistry was used to predict lymphomas with *TP53* mutations. The corresponding p53 immunohistochemistry staining for the lymphoma with the MI33I mutation and the lymphoma with wild-type *TP53* are given. (**c**) Overall patient survival based on the CD19 and *TP53* mutation statuses of lymphomas. *TP53* mutations were identified by gene sequencing of tumor DNA and predicted by *TP53* immunohistochemistry. WT, wild-type p53; TP53, tumor protein 53; −ve, negative; +ve, positive. p = 0.0003 (overall survival comparison between those with *TP53* mutant and wild-type *TP53* tumors).

Ninety-four DLBCL were included in the study. The patient demographics and clinical information are provided in Table 1. Sixteen DLBCL were CD19 negative based on a low expression of CD19 positive cells by flow cytometry (a clear visible shift in fluorescence consistent with reduced expression) based on the initial diagnostic assessment. A lack of CD19 positive cells in these tumors was confirmed using immunohistochemistry (Fig. 1a). The CD19 negative DLBCL were characterized further for other B cell lineage and lymphoma markers including CD20, PAX5, CD138, BCL2, BCL6, BCL10 and MUM1 using immunohistochemistry (Table 2). All CD19 negative lymphomas were positive for CD20, the majority was positive for PAX5, and two were positive for BCL2 and BCL6. A CD138 negative status for all tumors indicated that the CD19 negative cohort used were not plasma cell tumors^13^.

**Table 1:** Patient demographics and clinical characteristics of included individuals with DLBCL.

		CD19 negative (n = 16)*	CD19 positive (n = 78)**
Gender	Female	37%	40%
	Male	63%	60%
Age	<60	67%	67%
	≥60	33%	33%
Treatment	CHOP	100%	100%
	R-CHOP	31%	38%
PFS at 5 years		14%	34%
OS at 5 years		21%	54%

OS, overall survival; PFS, progression free survival; follow-up data available for 14 individuals (*) and 50 individuals (**).

**Table 2:** Summary of the B-cell and lymphoma marker distribution amongst CD19 and *TP53* subgroups.

Marker	CD19 negative mutant *TP53* (n = 13)	CD19 positive mutant *TP53* (n = 16)	CD19 positive wild-type *TP53* (n = 62)
BCL2	2/13 (15%)	3/16 (19%)	17/62 (27%)
BCL6	2/13 (15%)	4/16 (25%)	21/62 (34%)
BCL10	7/13 (54%)	7/16 (44%)	14/40 (35%)
CD20	13/13 (100%)	16/16 (100%)	61/62 (98%)
CD138	0/13 (0%)	0/16 (0%)	0/62 (0%)
MUM-1	2/13 (15%)	6/16 (38%)	16/40 (40%)
c-myc	7/13 (54%)	8/16 (50%)	13/62 (21%)
PAX5	10/13 (77%)	8/16 (50%)	53/62 (85%)

To determine if the irregular B-cell phenotype in CD19 negative lymphoma was associated with mutant *TP53*, all DLBCL were stained for p53 using immunohistochemistry and the *TP53* gene was sequenced (Fig. 1b). We found that 13/16 (81%) CD19 negative lymphomas had mutations in *TP53* whereas only 16/78 (21%) CD19 positive cases had *TP53* mutations. Thus, a higher proportion of *TP53* mutations occur in CD19 negative compared to CD19 positive lymphoma (p < 0.0001).

The lymphomas were further divided into 3 groups based on the CD19 status and the presence of *TP53* mutations. Of interest, irrespective of the CD19 status, tumors with mutant *TP53* had a poorer outcome compared to those with wild type *TP53* (median survival of 17 and 112 months respectively, p = 0.0003, hazard ratio 2.9, 95% CI 1.7–6.5) (Fig. 1c).

Mutant *TP53* is associated with poorer survival of DLBCL patients due in part to an association with increased function of the c-myc oncogene^5, 7, 9^. To determine if *TP53* mutations were associated with increased c-myc expression in our cohort, all lymphomas were stained with an antibody to c-myc (Table 2)^7^. Consistent with the above, we found that c-myc positive tumors were present in 50% of CD19 positive lymphomas with *TP53* mutations; 54% of CD19 negative lymphomas, but only 21% in tumors with wild-type *TP53* (p = 0.008, *TP53* mutant lymphoma (combined CD19 negative and positive cases) versus wild-type *TP53* cases).

An analysis of BCL10 and MUM1 was performed to determine if CD19 negative lymphomas had a germinal center (GC) or a non-germinal center-like phenotype^14^. Seventy-two cases were stained for BCL10 and MUM1: all CD19 negative lymphoma, and 56/78 CD19 positive lymphoma. Fifty-four percent (7/13) of the CD19 negative lymphoma cohort with *TP53* mutations (Table 2) was positive for BCL10 compared to 38% of CD19 positive lymphoma. Fifteen percent (n = 2/13) of the CD19 negative lymphoma cohort with *TP53* mutations (Table 2) was positive MUM-1 compared to 39% of the CD19 positive lymphoma cohort. The different frequencies of BCL10 and MUM1 positive cases were not significant between CD19 negative and positive lymphoma.

### Other B-cell irregularities are found in DLBCL with mutant *TP53*

To determine if *TP53* mutant DLBCL had other aberrantly expressed lineage markers, CD20 and PAX5 immunohistochemistry results were evaluated in CD19 positive lymphomas with *TP53* mutations in comparison to CD19 positive lymphomas with wild-type *TP53* (Table 2). No evidence was found that CD20 was lost on CD19 positive lymphomas with mutant *TP53*; however, 50% (n = 8/16) were negative for PAX5 compared with only 15% (n = 9/62) of DLBCL with wild-type *TP53* (p = 0.002). This result suggests aggressive lymphomas with mutant *TP53* have other irregular B-cell marker phenotypes. No differences were found between these lymphomas for BCL2, BCL6, and CD138 markers.

### CD19 negative lymphomas have an atypical distribution of *TP53* mutations

**Figure 2:**
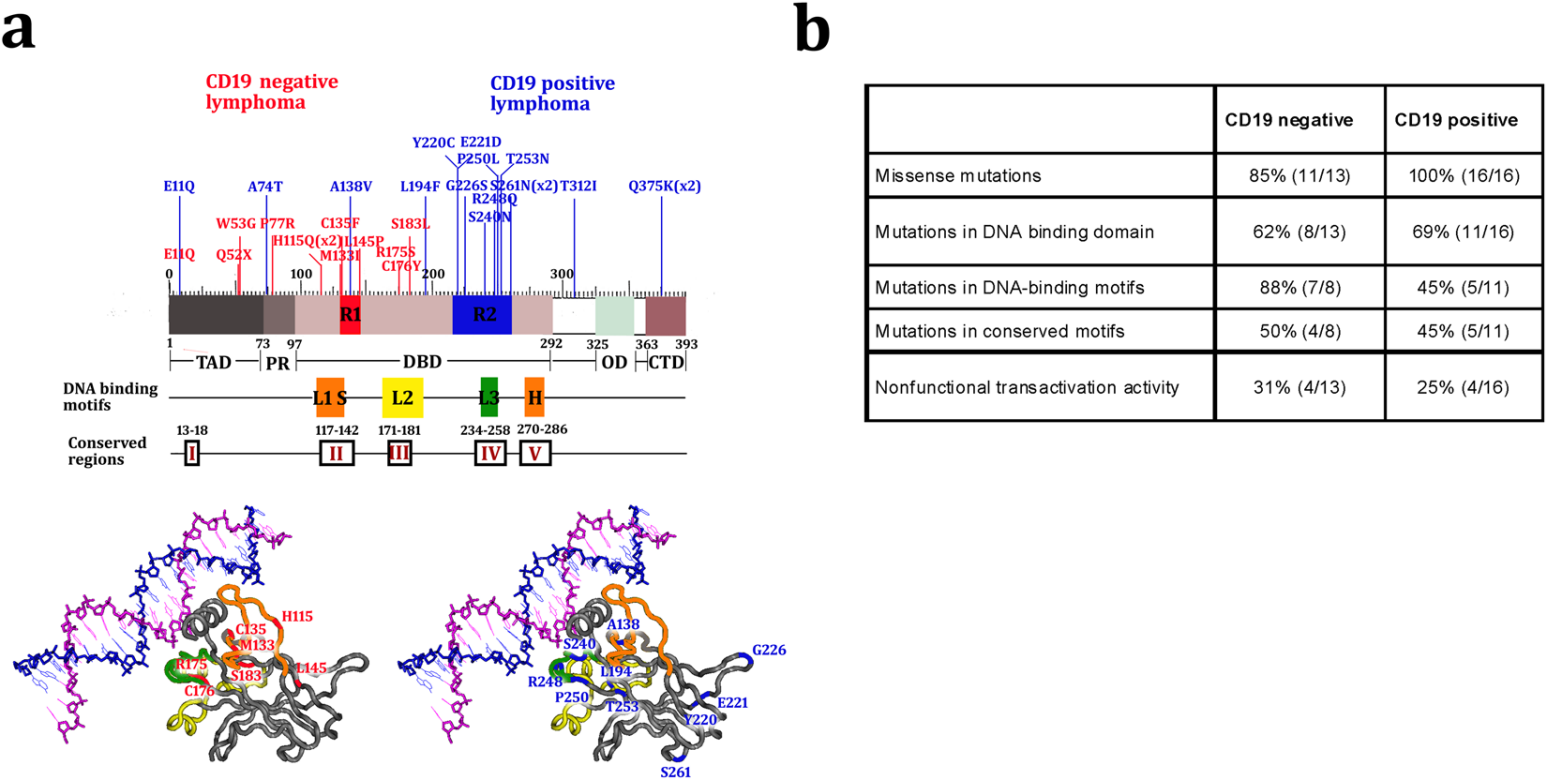
Atypical distribution of TP53 mutations in CD19 negative compared to CD19 positive B cell lymphomas. (**a**) Top panel: *TP53* mutation distribution column plot in CD19 negative (red columns) and CD19 positive (blue columns) DLBCL and their relation to p53 protein structure. TP53 mutations in DBD regions 1 and 2 (R1 and R2) targeted for comparison using RNAseq TCGA data are boxed in red and blue, respectively. The DNA binding loops of p53: LSH, L2 and L3 are boxed in orange, yellow and green, respectively. The location of highly conserved regions of the DNA binding domain are marked (I to V). Numbers indicate amino acid position. TAD, transactivation domain; PRR, proline-rich region; DBD, DNA-binding (core) domain; OD, tetramerization domain; CTD, regulatory domain at the extreme C-terminus. Bottom panel: position of *TP53* mutations within the 3D structure of p53 DNA-binding domain in CD19 negative (bottom left; highlighted in red) and CD19 positive (bottom right; highlighted in blue) DLBCL. Protein Data Bank code 1TSR file; chain B visualized by cn3d version 4.3^27^. (**b**) Structural and IARC mutation information for *TP53* mutants in CD19 negative and CD19 positive DLBCL (http://www-p53.iarc.fr/).

The nature of *TP53* mutations may provide clues as to the molecular events associated with the development of some cancer types^2^. Here we investigated the mutation spectrum in CD19 negative and CD19 positive DLBCL. Mutations detected were analyzed using the International Agency for Research on Cancer (IARC) database (http://www-p53.iarc.fr/). Overall, the mutation type was similar between the CD19 negative and CD19 positive cohorts with missense mutations being most frequent accounting for 85% and 100% of cases, respectively (Fig. 2). A nonsense mutation and a splice site mutation were identified in two CD19 negative DLBCL at Q52X and IVS4-2A > G, respectively.

There was however, a striking difference in spatial distribution of *TP53* mutations seen in CD19 negative compared to CD19 positive lymphomas. Notably, the *TP53* mutations identified in CD19 negative lymphomas were mainly located within the N-terminus of p53 and in the proximal region of the central core DNA-binding domain (DBD) with exons 4–5 most often mutated (Fig. 2a). Of the mutations located in the DBD: 88% (7/8) were located in DNA-binding motifs (DBM), four of these mutations were located in Loop 1 (L1) of the Loop-sheet-helix/LSH motif that interacts with the DNA major groove, and three mutations were in Loop 2 (L2). No mutations were located in L3 (codons 237–250) that enhances the binding affinity of p53 with the DNA helix (Fig. 2a and b)^15^. Four mutations were localized to highly conserved areas: two mutations in area II (codons 117–142) and two mutations in area III (codons 171–181). In contrast, in the CD19 positive cohort the majority of mutations were located in the distal part of the DBD, exons 6–7 (Fig. 2a). Only 45% (5/11) of mutations were inside the DBM with three located in L3 (Fig. 2a and b).

A comparison of the loss of function predictions using the IARC database did not reveal any differences in regard to mutations identified in CD19 positive and CD19 negative cases (Fig. 2b; http://www-p53.iarc.fr)^2^.

### Spatial distribution of *TP53* mutations alters expression of differentiation genes

Given the differences in mutation spectrum between CD19 positive and negative lymphomas and a tendency towards altered lineage marker expression, we asked whether different mutations might affect a generalized alteration in expression of differentiation genes. To test this, RNAseq data for tumors from The Cancer Genome Atlas (TCGA) with mutations analogous to those found in the CD19 negative lymphomas (region (R) 1 highlighted in Fig. 2a) were compared with mutations found in CD19 positive lymphomas (R2 highlighted in Fig. 2a). We found that 429 genes were differentially regulated (fold change cut off ≥1.5 or ≤−1.5 and p ≤ 0.05). These differentially expressed genes enriched for 13 GO-biological processes (p < 0.05). Interestingly, one of these over represented GO-biological processes included in developmental processes (GO:0032502), which encompasses genes involved in cell differentiation. To verify the involvement of these differentially expressed genes in cell differentiation geneset enrichment analysis was performed. We found that there was a significant enrichment for genes involved in differentiation (geneSetTest p = 0.002).

## Discussion

In this study, we showed *TP53* mutations were present in the majority of DLBCL with an irregular CD19 B-cell marker phenotype. These results suggest aggressive lymphomas with an irregular B-cell marker phenotype are enriched for *TP53* mutations. Based on gene expression differences *TP53* mutations present in CD19 negative lymphomas were associated with differentiation processes that could contribute to irregular B cell lineage marker expression. In mice without the p53 proline domain irregular B-cell phenotypes develop with age suggesting early changes to p53 lead to irregular B-cells^4^. Although this evidence is consistent with wild-type p53 playing a role in aiding correct B cell differentiation, in the current study we do not know when the *TP53* mutations occurred and thus were unable to determine if the *TP53* mutations were directly responsible for the irregular B-cell phenotype.

Growing evidence suggests that the pattern of *TP53* mutations varies amongst cancer types and may contribute to the tumor phenotype^6^. *TP53* mutations in CD19 negative lymphomas were distributed in the N-terminus of p53 in the proximal part of the DBD, and were more likely to be in the DBM in L1 and L2 regions. Previous studies including those in DLBCL have emphasized that missense mutations in the DBMs have a worse prognosis compared to missense mutations outside these motifs^6, 16^. Loss of transactivation capacity of p53 has been reported as the main determinant of the poor prognostic value of *TP53* in a number of tumors including those with inherited *TP53* mutations^2, 16, 17^. In CD19 positive lymphomas *TP53* mutations were distributed predominantly in the DBD distal to those in CD19 negative lymphomas and included mutations in L3. The location of mutations in CD19 positive lymphomas were more similar to that reported for *TP53* mutations in cancer in general (http://www-p53.iarc.fr/).

A limitation of the current study is that with the restricted number of CD19 negative lymphomas other factors, such as the treatment received, were not factored into the survival analyses. However, other studies of lymphomas have associated *TP53* mutations or loss of CD19 as factors associated with poorer patient survival^5, 6, 12^. The finding of this study, that CD19 negative lymphomas are enriched for *TP53* mutations, suggests *TP53* mutations may be the underlying factor associated with poorer patient outcome.

CD19 positive lymphomas with *TP53* mutations may have other irregularities in the B-cell phenotype. CD19 positive lymphomas with mutant p53 were more likely to have lost PAX5 expression. Loss of PAX5 contributes to leukemia and lymphoma with haplo-insufficiency of *PAX5* occurring in 32% of B-progenitor acute lymphoblastic leukemia^18^. Loss of PAX5 could be expected to create a more aggressive tumor. *In vitro* silencing of *PAX5* in mantle cell lymphoma led to decreased *TP53* expression and a more aggressive and drug resistant cell^19^. Mouse models lacking PAX5 in mature B-cells were prone to aggressive progenitor B-cell lymphomas^20^. CD19 is a downstream target of PAX5, so it is not immediately obvious how lymphomas can maintain CD19 expression without PAX5 function^21–23^. The analysis of PAX5 was limited to an immunohistochemistry analysis and although reduced there may be sufficient PAX5 present in the CD19 positive lymphomas classified as PAX5 negative to allow CD19 expression.

Overall our study demonstrates that *TP53* mutations are enriched in DLBCL with irregular expression of CD19 and PAX5. These findings suggest that the association between loss of B cell marker expression and *TP53* mutations may be relevant to those with relapsed disease following treatment with B cell targeted therapies. This has recently been shown in a case study, where a patient that experience a relapse post CD19 targeted therapy had CD19 negative disease at relapse^24^.

## Materials and Methods

### Case Selection

CD19 negative DLBCL (as determined by flow cytometry to have <10% positive cells as part of the initial diagnosis) cases were selected from biopsies taken between 1998–2015. Seventy-eight CD19 positive DLBCL were also selected from biopsies taken over the same period for comparison. Exclusion criteria included plasmablastic DLBCL and recurrent biopsies. Follow-up data was available for 64 individuals. This study was contacted with ethical approval from the New Zealand Health and Disability Ethics Committee and all methods were performed in accordance with the relevant guidelines and regulations. All study participants provided written and informed consent for study participation and acquisition of relevant clinical history.

### Immunohistochemistry

For immunohistochemical staining 4 µm sections from paraffin embedded tissues were subjected to heat mediated epitope-retrieval, and DAB chromogenic detection methods. B cell lymphoma 2, 6, and 10 (BCL2, BCL6, and BCL10), CD19, CD20, melanoma associated antigen (mutated) 1 (MUM1), PAX5, p53, and v-myc avian myelocytomatosis viral oncogene homolog (MYC) staining was performed. The primary antibodies and criteria for determining positive and negative tumors are given in Supplementary Table S1. Positive cells were counted at 400x magnification with at least 10 fields selected. The percent positive cells were calculated from the total number of cells counted (at least 500) by two blinded examiners.

### *TP53* mutation analysis

Tumor DNA was extracted from paraffin embedded tissues using the NucleoSpin FFPE RNA/DNA kit (Macherey-Nagel, Duren, Germany) according to the manufacturer’s instructions with each extraction including 3–10, 10 µm sections. *TP53* exons 1–11 including the intron-exon boundaries were amplified by PCR and sequenced by direct sequencing. Primer sequences are given in Supplementary Table S2.

### Statistical analyses

The frequency of *TP53* mutations amongst CD19 negative and CD19 positive lymphomas was compared using the Chi-square test. Kaplan Meier survival curves were compared between groups using the Log-rank test using SPSS version 22 software, with p values corrected for multiple comparisons using the Bonferroni correction. p < 0.05 was considered statistically significant.

### Retrieval of mutation and RNAseq data from TCGA datasets and differential gene expression analysis

Tumors containing missense and nonsense *TP53* mutations common to the position of the amino acids substituted in the CD19 negative and positive DLBCL respectively, were identified using cBioPortal (http://www.cbioportal.org/). Level 3 RNAseq data, processed using the RNAseq by Expectation-Maximization (RSEM) method^25^ and normalized within samples to a fixed upper quartile, was downloaded on October 20th 2015 (Supplementary Table S3). Detailed description of the processing protocol can be found in the TCGA open access FTP download directories (https://tcga-data.nci.nih.gov/tcgafiles/ftp_auth/distro_ftpusers/anonymous/tumor/). A list of differentially expressed genes in tumors with *TP53* mutations in the DBD (highlighted as R1 in Fig. 2a) common to the amino acid positions substituted in CD19 negative DLBCL (n = 6) versus *TP53* mutations in the DBD (highlighted as R2 in Fig. 2a) in CD19 positive DLBCL (n = 9) was generated using the limma-voom pipeline^26^. Nucleotide positions R175, C176, L194, Y220, R248 have been documented to be natural mutation hot-spots and hence were excluded from this analysis. Differentially expressed genes with a fold change of 1.5 and p value < 0.05 were used for pathway analyses. Pathway analyses were performed using PANTHER (www.pantherdb.org). A customized list of genes involved in differentiation of different cell lineages was downloaded from genesetdb (http://genesetdb.auckland.ac.nz/haeremai.html) (Supplementary Table S4). The genesetTest function from the “LIMMA” package (https://bioconductor.org/packages/release/bioc/html/limma.html) was used to analyze genes enriched within the customized differentiation geneset. All data analyses and visualizations were performed using the R statistical framework (https://www.r-project.org/).

## Electronic supplementary material

Supplementary tables
