# Supplementary material for: Tumor protein 53 mutations are enriched in diffuse large B-cell lymphoma with irregular CD19 marker expression
Source: Sci Rep. 2017 May 8;7:1566. doi: 10.1038/s41598-017-01800-6 (PMC5431468; doi:10.1038/s41598-017-01800-6)
Supplement: Supplementary file 1 — Supplementary tables [file 41598_2017_1800_MOESM1_ESM.pdf]

Tumor protein 53 mutations are enriched in diffuse large B-cell lymphoma with irregular CD19 marker expression

Authors and Affiliations

Marina Kazantseva, Noelyn A. Hung, Sunali Mehta, Imogen Roth, Ramona Eiholzer, Alison M. Rich, Benedict Seo, Margaret A. Baird, Antony W. Braithwaite, Tania L. Slatter

Supplementary Table S1: Primary antibodies and criteria for interpreting staining for immunohistochemistry analyses.

**Marker**	**Clone**	**Supplier**	**Staining interpretation (positive)**
BCL2	124	Dako	>30% [1]
BCL6	PG-B6p	Dako	>50% [1]
BCL10	151	Dako	>20% nuclear [2]
CD19	LE-CD19	Dako	>30%
CD20	L26	Dako	>30% [3]
CD138	B-A38	Cell Marque	>25%[4]
c-MYC	Ep121	Cell Marque	>40% [5]
MUM1	MRQ-8	Cell Marque	>40% [6]
PAX5	24	Cell Marque	>50% [1]
TP53	DO-7	Dako	>50% [7]

Dako, Glostrup, Denmark; Cell Marque Corporation, Rocklin, CA, U S A).

Supplementary Table S2: Primer sequences using to amplify *TP53* exons.

Exon	Forward Primers	Reverse primers
1	5’-CTTGTCATGGCGACTGTCCAG-3’	5’-CGAGAGCCCGTGACTCAGAGAG-3’
2	5’-CAGGTGACCCAGGGTTGGAAG-3’	5’-GCCTGCCCTTCCAATGGATG-3’
3	5’-CAGAGACCTGTGGGAAGCGA-3’	5’-CAGGTCCCCAGCCCTCCAGG-3’
4	5’-CAACGTTCTGGTAAGGACAAG -3’<br>5’- CGATATTGAACAATGGTTCA -3’	5’- GGCATTCTGGGAGCTTCATC -3’<br>5’- CATTGAAGTCTCATCCAAG -3’
5	5’-CTTGTGCCCTGACTTTCAACTCTG -3’<br>5’-GTGCAGCTGTGGGTTGATTCC -3’	5’-CTGCTTGTAGATGGCCATGG -3’<br>5’- GACCCTGGGCAACCAGCCCT -3’
6	5’-CGACAGGGCTGGTTGCCCAG -3’	5’-CTCCCAGAGACCCCAGTTG -3’
7	5’-CAAGGCGCACTGGCCTCATCTTG -3’	5’-CAGGCCAGTGTGCAGGGTGG -3’
8	5’-AATGGGACAGGTAGGACCTG-3’	5’-CTGAGGCATAACTGCACCCT-3’
9	5’-AGGGTGCAGTTATGCCTCAG -3’	5’-CTGGAAACTTTCCACTTGATAAC -3’

10	5'-GTAGCTAACTAACTTCA GAAC -3'	5'-CAGGCTAGGCTAAGCTATGATG -3'
11	5'-AACTCAGGTACTGTGTAT ATAC-3'	5'-CCTATGGCTTTCCAACCTAG-3'
12	5'-CAGACCCTCTCACTCA TG TG -3'	5'CTGACGCACAGGTATTGCAAG-3'

Supplementary Table S3. Tumor and mutation details on the *TP53* mutations common to the to the amino acid positions substituted in either CD19 negative or positive DLBCL.

Cancer Study	Sample ID	Amino Acid Change	Type	AA change position common to DLBCL with CD19 status
Breast Invasive Carcinoma (BRCA)	TCGA-A1-A0SK-01	M133K	Missense	Negative
Head and Neck Squamous Cell Carcinoma (HNSC)	TCGA-CV-7415-01			
Breast Invasive Carcinoma (BRCA)	TCGA-A2-A3XV-01	C135F	Missense	
Lung Adenocarcinoma (LUAD)	TCGA-55-7573-01			

Stomach Adenocarcinoma (STAD)	TCGA-CD-5800-01			
Brain Lower Grade Glioma (LGG)	TCGA-RY-A845-01	L145P	Missense	
Bladder Urothelial Carcinoma (BLCA)	TCGA-DK-A1AB-01	E221*	Nonsense	Positive
Breast Invasive Carcinoma (BRCA)	TCGA-A7-A13D-01			
Liver Hepatocellular Carcinoma (LIHC)	TCGA-CC-A7IG-01			
Lung Squamous Cell Carcinoma (LUSC)	TCGA-22-5492-01			
Stomach Adenocarcinoma (STAD)	TCGA-CG-4477-01	E221Afs*2	Frameshift	
Stomach Adenocarcinoma (STAD)	TCGA-BR-8683-01	G226Afs*21	Frameshift	

Head and Neck Squamous cell carcinoma (HNSC)	TCGA-CV-7432-01	S240Gfs*20	Frameshift	
Bladder Urothelial Carcinoma (BLCA)	TCGA-DK-A3IS-01	S240Kfs*24	Frameshift	
Breast Invasive Carcinoma (BRCA)	TCGA-GM-A2DI-01	S261*	Nonsense	

Supplementary Table S4. List of Gene ontologies and Pathway downloaded from genesetdb [8] used to make the customize list of genes involved in differentiation.

Class	Set Name	Source DB	Gene #
GO	glial cell differentiation (GO:0010001)	GO_BP	15
GO	regulation of neuron differentiation (GO:0045664)	GO_BP	16
Pathway	Keratinocyte Differentiation	Biocarta	37
GO	macrophage differentiation (GO:0030225)	GO_BP	11
GO	negative regulation of neuron differentiation (GO:0045665)	GO_BP	47
GO	T cell differentiation (GO:0030217)	GO_BP	24
GO	keratinocyte differentiation (GO:0030216)	GO_BP	45
GO	neuron differentiation (GO:0030182)	GO_BP	81
GO	positive regulation of macrophage derived foam cell differentiation (GO:0010744)	GO_BP	14
GO	regulation of cell differentiation (GO:0045595)	GO_BP	24

GO	B cell differentiation (GO:0030183)	GO_BP	39
GO	mammary gland epithelial cell differentiation (GO:0060644)	GO_BP	13
GO	positive regulation of B cell differentiation (GO:0045579)	GO_BP	10
GO	epithelial cell differentiation (GO:0030855)	GO_BP	40
GO	positive regulation of cell differentiation (GO:0045597)	GO_BP	23

References

1 Gualco, G., Natkunam, Y. & Bacchi, C. E. The spectrum of B-cell lymphoma, unclassifiable, with features intermediate between diffuse large B-cell lymphoma and classical Hodgkin lymphoma: a description of 10 cases. *Modern pathology : an official journal of the United States and Canadian Academy of Pathology, Inc* **25**, 661-674, doi:10.1038/modpathol.2011.200 (2012).

2 Merzianu, M. *et al.* Nuclear BCL-10 expression is common in lymphoplasmacytic lymphoma/Waldenstrom macroglobulinemia and does not correlate with p65 NF-kappaB activation. *Modern pathology : an official journal of the United States and Canadian Academy of Pathology, Inc* **19**, 891-898, doi:10.1038/modpathol.3800609 (2006).

3 Hans, C. P. *et al.* Confirmation of the molecular classification of diffuse large B-cell lymphoma by immunohistochemistry using a tissue microarray. *Blood* **103**, 275-282, doi:10.1182/blood-2003-05-1545 (2004).

4 Colomo, L. *et al.* Clinical impact of the differentiation profile assessed by immunophenotyping in patients with diffuse large B-cell lymphoma. *Blood* **101**, 78-84, doi:10.1182/blood-2002-04-1286 (2003).

5 Green, T. M. *et al.* Immunohistochemical double-hit score is a strong predictor of outcome in patients with diffuse large B-cell lymphoma treated with rituximab plus cyclophosphamide, doxorubicin, vincristine, and prednisone. *Journal of clinical oncology : official journal of the American Society of Clinical Oncology* **30**, 3460-3467, doi:10.1200/JCO.2011.41.4342 (2012).

6 Anderson, J. J. *et al.* Immunophenotyping of diffuse large B-cell lymphoma (DLBCL) defines multiple sub-groups of germinal centre-like tumours displaying different survival characteristics. *International journal of oncology* **35**, 961-971 (2009).

7 Xu-Monette, Z. Y. *et al.* Mutational profile and prognostic significance of TP53 in diffuse large B-cell lymphoma patients treated with R-CHOP: report from an International DLBCL Rituximab-CHOP Consortium Program Study. *Blood* **120**, 3986-3996, doi:10.1182/blood-2012-05-433334 (2012).

8 Araki, H., Knapp, C., Tsai, P. & Print, C. GeneSetDB: A comprehensive meta-database, statistical and visualisation framework for gene set analysis. *FEBS open bio* **2**, 76-82, doi:10.1016/j.fob.2012.04.003 (2012).
